# Supplementary material for: Informal workers’ perceptions of retirement planning in developing countries
Source: PLoS One. 2025 Apr 15;20(4):e0321214. doi: 10.1371/journal.pone.0321214 (PMC11999124; doi:10.1371/journal.pone.0321214)
Supplement: S2 Appendix — (DOCX) [file pone.0321214.s002.docx]

**APPENDIX: SURVEY INSTRUMENT**

**Section 1: Demographic Information**

| 1 | Name: |  |
| --- | --- | --- |
| 2 | Age: |  |
| 3 | Location: |  |
| 4 | Gender: |  |
| 5 | Family Size: |  |
| 6 | Personal Income range? |  |
| 7 | Family Income range? |  |
| 8 | Vendor only or Farmer and Vendor? |  |
| 9 | Number of people working in the family? |  |
| 10 | What work do other family members do? |  |
| 11 | Duration as a market vendor? |  |
| 12 | Occupation prior to a vendor? |  |

**Section 2: Savings related questions**

Question 1: Do you save? (Y/N)

*If yes to question 1, then*

A. How much do you save daily/weekly/annually?

B. Where do you usually save?

C. What avenues of savings are you aware of?

___________________________________________________________________________

D. Are you aware of any savings scheme from FNPF?

E. Are you aware of the benefits of savings with FNPF?

*If no to question 1, then*

A. Are you willing to save?

*If yes to question A, then*

I. How much are you willing to save?

II. Where would you be willing save?

III. Are you aware of any savings scheme from FNPF?

IV. Are you aware of the benefits of savings with FNPF?

*If no to question A, then*

I. Are you aware of the benefits of savings (in general)?

*If yes to question I above, then*

a. Are you aware of any savings scheme from FNPF?

b. Are you aware of the benefits of savings with FNPF?

Question 2: Do you think there should be a tailor-made savings scheme for market vendors? If so, why do you think so?

|  |
| --- |
|  |
|  |

Question 3: What are your perceptions towards the FNPF?

A. If positive/negative/neutral, why?

|  |
| --- |
|  |
|  |
|  |
|  |

Question 4: Would you recommend your children or relatives or anyone to become a market vendor?

____________________________________________________________________________________________________________________________________________________________________________________________________________________________________________________________________________________________________________________________________________________________________________________________________________________________________________________________________________________________________________
