# Supplementary material for: Informal workers’ perceptions of retirement planning in developing countries
Source: PLoS One. 2025 Apr 15;20(4):e0321214. doi: 10.1371/journal.pone.0321214 (PMC11999124; doi:10.1371/journal.pone.0321214)
Supplement: Attachment — Submitted filename: Referee Report_ZL_20240113.docx [file pone.0321214.s003.docx]

**REFEREE REPORT**

**PONE-D-23-21438: Informal Workers’ Perceptions of Retirement Planning in Development Countries**

**Comments to the Authors**

This paper examines whether and to what extent informal sector workers (i.e., vegetable market venders, or farmers) in Fiji engage in retirement savings—primarily by evaluating their awareness and access to Fiji National Provident Fund (FNPF). In doing so, the authors deploy several empirical estimation strategies including the OLS regression, correlation analysis, and principal component analysis on a first-hand survey (case studies?) of 71 respondents. The authors find that most respondents regarded FNPF in a positive light, with 60-70% of the respondents being aware of the FNPF scheme and its benefits. Yet, 94% of them still preferred an alternative pension scheme that is better tailored for them. The authors also find that age, gender, years of experience (duration) in the current job, and availability of other venues for savings strongly predicted the likelihood of being aware of FNPF schemes.

While I see the value in the authors’ introducing a primary dataset on an underexplored group of informal agricultural sector workers in Fiji, I find several limitations in the overall framing (i.e., motivation for examining FNPF) of the research questions, estimation strategies, and interpretation of the results. Moreover, the authors equate ‘awareness’ of a government-sponsored pension/superannuation scheme (i.e., FNPF) with ‘financial literacy’—when these two are discussed as two distinct concepts in the current literature. I discuss my main concerns below, in no particular order.

**MAIN REMARKS**

- **Weak framing of the research questions:**
  - The framing of the paper, detailed in the Introduction, is weak. The authors start the paper with the need for an increase of world’s food production to fight global poverty and population growth. It is strange for me to see the authors linking ‘poverty’ and ‘population growth’ to ‘food production’. Poverty arises not because of unavailability of food (the global stocks) but primarily due to the unequal distribution/access/affordability of food around the world.
  - The authors also use ‘(the need to boost) food production’ as a reason to care about the ‘welfare of food suppliers’. This is not wrong, but is this necessary? Without making these jumps, I feel like the paper can just start with a need to improve welfare for informal sector workers—who are often excluded from social safety nets and government protection programs, just based on the right to decent work and job security.
  - The authors’ justifying the need for FNPF and other retirement savings schemes of ag farmers based on the incentives they give for food production (i.e., increasing productivity) is awkward because retirement savings essentially provide annuities *after* retiring. Do retirement savings really provide ag farmers incentives to be more productive *during* their working years? The rest of the paper talks about retirement financial planning, so I am not sure whether this ‘boosting production’ framing is effective.
- **Lacking flow**: The paper often lacks flow of narrative. For instance, the authors note in lines 66-69 in the Introduction that the main elements that contributed to the demise of the ag sector (and its production levels) were “property rights issues, preferential agreements, natural disasters, rural-urban migration, and lack of diversification.” None of these point to the lack of welfare or retirement benefits. So, if the authors were to start with the ‘need to boost food production’, the authors should be addressing existing government policies that deal with any of the key elements above—and not a pension scheme/retirement financing. Following the list of key elements that deterred production in the ag sector, the authors say that they study FNPF in this study (lines 71-73). At the moment, it is not clear why the discussion of the declining production in the ag sector calls for a need to study FNPF. How does this superannuation fund address the key elements of the demise?
  - Another instance can be found in the Lit Review section. The subsections are fundamentally related, but the authors don’t make enough effort to point out any connections.
- **Overall, motivation and justification for the authors’ exploration of FNPF (detailed in the paper) need a lot more polishing.**
- **There is no justification for several methods used in the paper.** For instance, what’s the value of using principal components analysis and regressions? **Data:** The process through which the authors create variables off the case studies (these are not surveys, right?) should also be more clearly stated.

**COMMENTS ON SPECIFIC SECTIONS**

Introduction

- **The scope of the paper can be made clearer**. For instance, the authors state that the paper address literature on ‘nest egg savings options. But isn’t the paper specifically centering on a type of contributory pension (Fiji National Provident Fund, or FNPF) and no other general saving schemes for non-retirement purposes? Why not just define the scope as looking at pension—and the extent to which ag farmers in Fiji rely on it?
- **More background info on FNPF is needed in the introduction**: FNPF is not mandatory (line 31, 56), but what does that mean? FNPF seems to be a contributory pension scheme where employers match the workers’ contribution amounts, and this is mandatory for formal sector workers. For informal sector workers (i.e., ag farmers) for whom FNPF is not mandatory, what happens if an informal sector worker decides to contribute to FNPF? Does his/her employer (in the informal sector) need to match the amount? Or are they all considered self-employed workers?
  - On a related note, what are the general retirement savings patterns in the Fiji informal sector? Any statistics or prior literature? What is the usual share of informal sector workers that rely on FNPF?
  - In the introduction, there is no background on retirement savings patterns of individuals in Fiji, or FNPF. There is one paragraph of the background on FNPF (lines 223-234) in the Lit Review section—but it comes way too late. It may be better to put these earlier, as a part of the Introduction
- Definition of informal workers seems incomplete (line 51-52). What about government protections or tax or retirement benefits eligibility?
- It may be helpful for general readers to define concepts such as ‘nest egg savings’ or ‘superannuation’ once in the introduction (lines 28, 30, 54).
- Going back to the awkwardness of justifying the need for FNPF and other retirement savings schemes of ag farmers based on the incentives for food production (i.e., increasing productivity), this comes back to bite in lines 57-64. In discussing policy alternatives to FNPF, the authors discuss policy alternatives to boost food production—including export subsidies. Isn’t the main purpose of FNPF or any other pension schemes to promote welfare and retirement preparedness?
  - Even if the authors decide to keep the narrative linking FNPF to boosting food production/productivity, the discussion of policy alternatives is very limited in the paper (lines 57-64) and requires more nuanced descriptions. There exist several micro-level policies that directly benefit workers—such as living subsidies, childcare subsidies, or even food vouchers.
- Lines 74-81: The specific survey Qs seem to ask whether workers utilize FNPF account, and to what extent,, and characteristics that predict FNPF usage/awareness. The 3^rd^ question (line 78-79) should be more carefully worded. The authors seem to estimate determinants that ascertain ag farmers’ probability/likelihood of being aware of FNPF. This is the outcome variable the authors use. The extent of awareness and the extent of accessibility are two different concepts.
- I see another stretch of an argument when the authors link the value of ‘financial planning’ (at an individual level) to ‘UN’s sustainable development goals or reducing poverty or reducing inequalities (for whom?)’.
- Lines 86-93 should probably go to the discussion/conclusion section. The authors talk about implications of findings here, but they haven’t described their findings yet.

Literature Review/Background

- **Overall:** The background section has too many subsections without any narrative cutting through them. They are fundamentally related, but the authors don’t make enough efforts to point out any connections.
- **RE Informal Sector and Financial Inclusion**: Relevant studies on informality and retirement preparedness that are the authors missed include the following:
  - Ginneken W. Social Security for the informal sector: a new challenge for the developing countries. Int Soc Secur Rev. 2002;52:49-69. https://doi. org/10.1111/1468-246X.00033
  - Hu YW, Stewart F. Pension coverage and informal sector workers: international experiences. OECD Publishing, 2009. Available from: <https://ideas.repec.org//p/oec/dafaab/31-en.html>
  - Lee, Z., Aguila, E., & Wong, R. (2023). Determinants of retirement of formal and informal sector workers in Mexico: the role of health and economic security. salud pública de méxico, 65(5), 434-445. <https://doi.org/10.21149/15178>
  - Onyango C, Olunga C, Oleche MO. Attitude of informal sector workers towards saving for retirement. IJNRIS. 2016;3(4):12-9 Available from: https://www.noveltyjournals.com/upload/paper/ ATTITUDES%20OF%20INFORMAL%20SECTOR-752.pdf
  - Tran C, Jung J. Transfers and labor market behavior of the elderly in developing countries: theory and evidence from Vietnam. New York: 2009 <https://doi.org/10.2139/ssrn.1147649>
- **RE Informal Sector and Financial Inclusion** **cont.:** There is a jump in logic in line 102. Up to this line, the authors discuss that informal sector research is limited. But why is financial inclusion suddenly brought up here? For instance, are informal labor characterized by financial exclusion (from govt programs? retirement financial under-preparedness?)? Also, the citations included in line 102 and onward are no longer about the informal sector. It is more about the value of financial inclusion generally. This section needs to be woven better. Why discuss informality and financial inclusion together? How do the two relate?
- **RE Financial Planning:** This section talks about financial planning generally and provides some details on superannuation (lines 121-). But given the overall topic of this paper (on informal sector workers), the authors should still tie this section to informal sectors. For instance, in what ways are superannuation schemes applied to informal workers? There is another sudden jump in line 140. Why the sudden discussion of retirement planning—when the prior discussions of financial planning in this section did not mention retirement planning at all?
- **RE Financial Planning cont.:** It is not clear why the authors discuss the link between retirement planning and financial literacy at length in this section (lines 140-). The authors need to provide clearer description as to why they are emphasizing financial literacy. For instance, are the authors empirically measuring financial literacy in their estimation? The authors go on to list determinants of financial literacy. These distract the readers. Are these drivers measured in the analysis? If yes, the authors should state this.
- **RE Superannuation in Developing Countries:** This section summarizes findings on several different aspects of pension, from (1) lack of participation or access to pension in Indonesia, to (2) effect of pension on well-being. I am afraid these studies are summarized one after another without a clear rationale. I would say, for this paper, the main thing I need to know from the existing literature is how superannuation contributes to retirement planning or retirement financial security. OR how superannuation boosts productivity of participating workers. More importantly, the authors seem to discuss the pensions without delineating between different types of pension (annuity vs lump sum provision, contributory, non-contributory). The effects of pension on retirement planning, for example, depends on the pension type. So the authors should make efforts to acknowledge the distinctions.
- **RE other investment opportunities for farmers:** Another floating section. This section can be woven together with the first background section (informal sector and financial planning), to show that farmers have alternatives for retirement savings: FNPF, or these other investment opportunities. Also, the authors need to better describe how this information is going to be used in their study.

Data/Methods

- Case studies🡪 survey Qs: The process through which the authors create variables off the case studies (these are not surveys, right?) should also be more clearly stated.
- Selection bias in the sample: It is unclear how the authors deal with selection bias in their survey/case studies. Line 248 states that sample is drawn to balance gender and ethnicity. But was the sample chosen at random (stratified based on gender and ethnicity)?
- The paper needs a description of how the variables are formulated, from what questions.
- RE estimation (regression, shown in the formula 1)
  - The outcome variables need to be better explained. What is the difference between FNPF-1 and FNPF-2? What are the ‘benefits’ of which respondents are aware in FNPF-2? Are these the expected benefits farmers are going to collect (therefore testing the respondents’ ability to calculate their expected benefits)?
  - Lines 277-279 suggest that the authors did ask the respondents about all means of retirement savings –in addition to FNPF. This point should be clarified earlier.
- **Sample size** **power issue**: The authors utilize a sample size of 71 respondents. This may be too small to generate statistically robust results for a regression with 8 regressors(independent variables). I suggest the authors conduct a power analysis to ascertain the minimum a-priori sample size for the regression (Faul et al. 2009). Using an online calculator, the minimum sample size the authors need for a regression with 8 regressors (with a moderate effect size of 0.15 and minimum statistical power level 0.8 (standard)) is 108 respondents.
  - <https://www.danielsoper.com/statcalc/calculator.aspx?id=1>
  - Faul, F., Erdfelder, E., Buchner, A., & Lang, A. G. (2009). Statistical power analyses using G* Power 3.1: Tests for correlation and regression analyses. Behavior research methods, 41(4), 1149-1160.

Findings

- It would be great if the authors could show a table of descriptive stats.
- RE “94% of respondents prefer a tailor-made retirement plan”🡪 This statement does not really add to the paper. What are the plan specifics that they desire? To start discussing these, the authors should provide a more comprehensive and critical evaluation of the FNPF in light of its (non)coverage of informal sector workers. What are the FNPF’s shortcomings, and are these what the respondents want in the ‘tailored plan’?
- RE line 332-333 “This contrasts the duration the person has been a market vendor.” This needs to be explained better.
- RE lines 333-335 “Other savings avenues have a positive effect….financial literacy enables one to be aware of FNPF’s services.” This is a possibility but not the literacy is not the only facilitator that incentivizes a farmer to have other savings. The bank accounts may be a joint account for the family. It could just mean the respondents are wealthier and are in need of a secure means to safeguard their finances.
- **Interpretation of the findings in Table 3 and Table 4:**
  - The points of convergence and divergence in terms of the effects of the drivers in Table 3 and Table 4 should be explained. For instance, why is gender a significant predictor for awareness of the FNPF benefits (table 4) but not general awareness of FNPF (table 3)?
  - Line 341-342: While gender is a weak predictor of awareness of the pension schemes, the sample doesn't necessarily suffer from 'lack of awareness of FNPF' generally. The summary stats showed that 60% know FNPF and nearly 70% are aware of the benefits of FNPF right? The authors need to interpret findings more carefully.
  - From the earlier description in formula (1), AVNE was coded as a categorical variable (saving venues). But in Table 3 and Table 4, I only see one variable AVNE in the regression. How is this possible?
- What's the rationale behind doing multiple estimations--correlation, regression, principal component analyses? Rationales are not given in the paper.

Conclusion

- Lines 400-401: The fact that 94% of the respondents prefer ‘tailored pension’ deserves more elaboration. What kinds of design improvements are necessary? To make such recommendations, the authors need to critically evaluate the shortcomings of FNPF (for informal workers/farmers) in the background section.
- The implications of the findings often do not align with their findings. For instance, in lines 404-405, the authors state that “FNPF needs to create more awareness for membership.” However, the awareness of FNPF in the sample was high—approximately 70%. This is quite high, compared to the level of understanding of other pension programs in other countries. I cannot agree that the lack of awareness is one of the main findings of this paper.
- While financial literacy is indeed an important component of retirement planning (and a hot topic these days), I am not sure whether there is value in emphasizing the role of financial literacy this much in the paper. The authors are not really tackling financial literacy in this paper, based on the ways financial literacy is defined and measured and studied in the recent literature. ‘Awareness' and ‘financial literacy’ are two different concepts.

**REFERENCES**

- Faul, F., Erdfelder, E., Buchner, A., & Lang, A. G. (2009). Statistical power analyses using G* Power 3.1: Tests for correlation and regression analyses. Behavior research methods, 41(4), 1149-1160.
- Ginneken W. Social Security for the informal sector: a new challenge for the developing countries. Int Soc Secur Rev. 2002;52:49-69. https://doi. org/10.1111/1468-246X.00033
- Hu YW, Stewart F. Pension coverage and informal sector workers: international experiences. OECD Publishing, 2009. Available from: <https://ideas.repec.org//p/oec/dafaab/31-en.html>
- Lee, Z., Aguila, E., & Wong, R. (2023). Determinants of retirement of formal and informal sector workers in Mexico: the role of health and economic security. salud pública de méxico, 65(5), 434-445. <https://doi.org/10.21149/15178>
- Onyango C, Olunga C, Oleche MO. Attitude of informal sector workers towards saving for retirement. IJNRIS. 2016;3(4):12-9 https://www.noveltyjournals.com/upload/paper/ ATTITUDES%20OF%20INFORMAL%20SECTOR-752.pdf
- Tran C, Jung J. Transfers and labor market behavior of the elderly in developing countries: theory and evidence from Vietnam. New York: 2009 https://doi.org/10.2139/ssrn.1147649
