# Supplementary material for: Informal workers’ perceptions of retirement planning in developing countries
Source: PLoS One. 2025 Apr 15;20(4):e0321214. doi: 10.1371/journal.pone.0321214 (PMC11999124; doi:10.1371/journal.pone.0321214)
Supplement: Attachment — Submitted filename: Reviewer Response Document.docx [file pone.0321214.s006.docx]

**Reviewer Response Document**

| Comments | Authors Response | Changes |
| --- | --- | --- |
| Reviewer 1 |  |  |
| The paper should not be published as it is because of inconsistent use of capitalization make the paper unintelligible. Financial planning and other terms are sometimes capitalized and sometimes not capitalized. These mistakes are critical mistakes in grammar.  Even more frustrating is that the authors have a unique opportunity to describe the Fiji pension system within the context of the Fji labor market. However the authors assume readers know the basic facts about Fiji. The section on the superannuation scheme is completely impenetrable. The authors should imagine sitting in a room of intelligent pension experts and seek to describe the system. After reading this paper carefully I absolutely have no idea what the Fiji pension system is like.  The authors also states they have established causality between financial literacy and subjective willingness to contribute to the Fiji system. But this is not made clear.  We always need papers on the world‘s pension systems and the inclusion of informal workers into social insurance schemes is probably the most important issue facing modern market economies. But this paper fails at describing, even the simplest terms, with the system is in Fiji. So frustrating. I would love to know about the Fiji superannuation schemes and policies that would bring in informal sector workers into the schemes. | We thank the reviewer for this comment. This has been addressed throughout the paper.  We thank the reviewer for this comment.  We have described Fiji’s superannuation fund in detail and incorporated it into the introduction section.  We thank the reviewer for this comment.  Our causality between financial literacy and willingness to save is based on 83.1% of vegetable market vendors saving in banks only. While 62.3% know of the option to save with FNPF, and 67.6% know the benefits of savings with FNPF, it is strange that 83.1% save in banks. The underlying reason is the lack of motivation to adopt FNPF as a savings option. This can be down to the fact that it is easier to save in a bank than with FNPF. Thus, the FNPF needs to make a tailor-made pension contribution scheme for vegetable market vendors, farmers and those in the informal sector.  We have described Fiji’s superannuation fund in detail and incorporated it into the introduction section as per the comments and changes above. | Throughout the manuscript.  FNPF is a compulsory superannuation scheme for local employees in the formal sector, where employers and employees are required to make 10% and 8% contributions of cash emoluments, respectively. During COVID-19, the government reduced these contributions to 5% respectively between 1 April 2020 and 31 December 2021. These contributions were raised to 6%, respectively, in 2022 and 7% in 2023. The 10% and 8% contributions were normalized from 1 January 2024 (PWC, 2024). The savings benefits with FNPF include retirement and pre-retirement benefits such as housing, medical, education, funeral, and unemployment assistance. The fund allocates members' contributions to the Preserved Account and General Account. The Preserved Account accumulates 70% of all contributions, while the remaining 30% is accounted for in the General Account. All the pre-retirement benefits are distributed from the General Account except for housing. On a member's purchase of their first property, they are entitled to all the contributions in the general account and 30% from the Preserved Account. Those working in the informal sector, self-employed and working overseas can contribute to the fund as voluntary members with a $10 opening deposit and a minimum of $10 deposit per transaction. Payments in the Voluntary membership can be made through bank transfers, payments at FNPF outlets, payments at Post Fiji (postal service providers with offices in diverse locations) outlets and mobile payments [55]. Altfest [23] states that the most common areas of interest to an individual in terms of PFP are tax planning, cash flow planning, investments, risk management, retirement planning, and estate planning. All these areas correspond in one way or another to the benefits provided by FNPF.  While the previous statistics show above-average awareness, it is strange that 83.1% save in banks. The underlying reason is the lack of motivation to adopt FNPF as a savings option. This can be down to the fact that it is easier for vegetable market vendors to save in a bank than with FNPF. Thus, the FNPF needs to make a tailor-made pension contribution scheme for vegetable market vendors, farmers and those in the informal sector. |
| Reviewer 2  **Weak framing of the research questions:**  The framing of the paper, detailed in the Introduction, is weak. The authors start the paper with the need for an increase of world’s food production to fight global poverty and population growth. It is strange for me to see the authors linking ‘poverty’ and ‘population growth’ to ‘food production’. Poverty arises not because of unavailability of food (the global stocks) but primarily due to the unequal distribution/access/affordability of food around the world.  The authors also use ‘(the need to boost) food production’ as a reason to care about the ‘welfare of food suppliers’. This is not wrong, but is this necessary? Without making these jumps, I feel like the paper can just start with a need to improve welfare for informal sector workers—who are often excluded from social safety nets and government protection programs, just based on the right to decent work and job security.  The authors’ justifying the need for FNPF and other retirement savings schemes of ag farmers based on the incentives they give for food production (i.e., increasing productivity) is awkward because retirement savings essentially provide annuities *after* retiring. Do retirement savings really provide ag farmers incentives to be more productive *during* their working years? The rest of the paper talks about retirement financial planning, so I am not sure whether this ‘boosting production’ framing is effective.  **Lacking flow**: The paper often lacks flow of narrative. For instance, the authors note in lines 66-69 in the Introduction that the main elements that contributed to the demise of the ag sector (and its production levels) were “property rights issues, preferential agreements, natural disasters, rural-urban migration, and lack of diversification.” None of these point to the lack of welfare or retirement benefits. So, if the authors were to start with the ‘need to boost food production’, the authors should be addressing existing government policies that deal with any of the key elements above—and not a pension scheme/retirement financing. Following the list of key elements that deterred production in the ag sector, the authors say that they study FNPF in this study (lines 71-73). At the moment, it is not clear why the discussion of the declining production in the ag sector calls for a need to study FNPF. How does this superannuation fund address the key elements of the demise?  Another instance can be found in the Lit Review section. The subsections are fundamentally related, but the authors don’t make enough effort to point out any connections.  **Overall, motivation and justification for the authors’ exploration of FNPF (detailed in the paper) need a lot more polishing.**  **There is no justification for several methods used in the paper.** For instance, what’s the value of using principal components analysis and regressions? **Data:** The process through which the authors create variables off the case studies (these are not surveys, right?) should also be more clearly stated.  **COMMENTS ON SPECIFIC SECTIONS**  Introduction  **The scope of the paper can be made clearer**. For instance, the authors state that the paper address literature on ‘nest egg savings options. But isn’t the paper specifically centering on a type of contributory pension (Fiji National Provident Fund, or FNPF) and no other general saving schemes for non-retirement purposes? Why not just define the scope as looking at pension—and the extent to which ag farmers in Fiji rely on it?  **More background info on FNPF is needed in the introduction**: FNPF is not mandatory (line 31, 56), but what does that mean? FNPF seems to be a contributory pension scheme where employers match the workers’ contribution amounts, and this is mandatory for formal sector workers. For informal sector workers (i.e., ag farmers) for whom FNPF is not mandatory, what happens if an informal sector worker decides to contribute to FNPF? Does his/her employer (in the informal sector) need to match the amount? Or are they all considered self-employed workers?  On a related note, what are the general retirement savings patterns in the Fiji informal sector? Any statistics or prior literature? What is the usual share of informal sector workers that rely on FNPF?  In the introduction, there is no background on retirement savings patterns of individuals in Fiji, or FNPF. There is one paragraph of the background on FNPF (lines 223-234) in the Lit Review section—but it comes way too late. It may be better to put these earlier, as a part of the Introduction  Definition of informal workers seems incomplete (line 51-52). What about government protections or tax or retirement benefits eligibility?  It may be helpful for general readers to define concepts such as ‘nest egg savings’ or ‘superannuation’ once in the introduction (lines 28, 30, 54).  Going back to the awkwardness of justifying the need for FNPF and other retirement savings schemes of ag farmers based on the incentives for food production (i.e., increasing productivity), this comes back to bite in lines 57-64. In discussing policy alternatives to FNPF, the authors discuss policy alternatives to boost food production—including export subsidies. Isn’t the main purpose of FNPF or any other pension schemes to promote welfare and retirement preparedness?  Even if the authors decide to keep the narrative linking FNPF to boosting food production/productivity, the discussion of policy alternatives is very limited in the paper (lines 57-64) and requires more nuanced descriptions. There exist several micro-level policies that directly benefit workers—such as living subsidies, childcare subsidies, or even food vouchers.  Lines 74-81: The specific survey Qs seem to ask whether workers utilize FNPF account, and to what extent,, and characteristics that predict FNPF usage/awareness. The 3^rd^ question (line 78-79) should be more carefully worded. The authors seem to estimate determinants that ascertain ag farmers’ probability/likelihood of being aware of FNPF. This is the outcome variable the authors use. The extent of awareness and the extent of accessibility are two different concepts.  I see another stretch of an argument when the authors link the value of ‘financial planning’ (at an individual level) to ‘UN’s sustainable development goals or reducing poverty or reducing inequalities (for whom?)’.  Lines 86-93 should probably go to the discussion/conclusion section. The authors talk about implications of findings here, but they haven’t described their findings yet.  Literature Review/Background  **Overall:** The background section has too many subsections without any narrative cutting through them. They are fundamentally related, but the authors don’t make enough effort to point out any connections.  **RE Informal Sector and Financial Inclusion**: Relevant studies on informality and retirement preparedness that are the authors missed include the following:  Ginneken W. Social Security for the informal sector: a new challenge for the developing countries. Int Soc Secur Rev. 2002;52:49-69. https://doi. org/10.1111/1468-246X.00033  Hu YW, Stewart F. Pension coverage and informal sector workers: international experiences. OECD Publishing, 2009. Available from: <https://ideas.repec.org//p/oec/dafaab/31-en.html>  Lee, Z., Aguila, E., & Wong, R. (2023). Determinants of retirement of formal and informal sector workers in Mexico: the role of health and economic security. salud pública de méxico, 65(5), 434-445. <https://doi.org/10.21149/15178>  Onyango C, Olunga C, Oleche MO. Attitude of informal sector workers towards saving for retirement. IJNRIS. 2016;3(4):12-9 Available from: https://www.noveltyjournals.com/upload/paper/ ATTITUDES%20OF%20INFORMAL%20SECTOR-752.pdf  Tran C, Jung J. Transfers and labor market behavior of the elderly in developing countries: theory and evidence from Vietnam. New York: 2009 <https://doi.org/10.2139/ssrn.1147649>  **RE Informal Sector and Financial Inclusion** **cont.:** There is a jump in logic in line 102. Up to this line, the authors discuss that informal sector research is limited. But why is financial inclusion suddenly brought up here? For instance, are informal labor characterized by financial exclusion (from govt programs? retirement financial under-preparedness?)? Also, the citations included in line 102 and onward are no longer about the informal sector. It is more about the value of financial inclusion generally. This section needs to be woven better. Why discuss informality and financial inclusion together? How do the two relate?  **RE Financial Planning:** This section talks about financial planning generally and provides some details on superannuation (lines 121-). But given the overall topic of this paper (on informal sector workers), the authors should still tie this section to informal sectors. For instance, in what ways are superannuation schemes applied to informal workers? There is another sudden jump in line 140. Why the sudden discussion of retirement planning—when the prior discussions of financial planning in this section did not mention retirement planning at all?  **RE Financial Planning cont.:** It is not clear why the authors discuss the link between retirement planning and financial literacy at length in this section (lines 140-). The authors need to provide clearer description as to why they are emphasizing financial literacy. For instance, are the authors empirically measuring financial literacy in their estimation? The authors go on to list determinants of financial literacy. These distract the readers. Are these drivers measured in the analysis? If yes, the authors should state this.  **RE Superannuation in Developing Countries:** This section summarizes findings on several different aspects of pension, from (1) lack of participation or access to pension in Indonesia, to (2) effect of pension on well-being. I am afraid these studies are summarized one after another without a clear rationale. I would say, for this paper, the main thing I need to know from the existing literature is how superannuation contributes to retirement planning or retirement financial security. OR how superannuation boosts productivity of participating workers. More importantly, the authors seem to discuss the pensions without delineating between different types of pension (annuity vs lump sum provision, contributory, non-contributory). The effects of pension on retirement planning, for example, depends on the pension type. So the authors should make efforts to acknowledge the distinctions.  **RE other investment opportunities for farmers:** Another floating section. This section can be woven together with the first background section (informal sector and financial planning), to show that farmers have alternatives for retirement savings: FNPF, or these other investment opportunities. Also, the authors need to better describe how this information is going to be used in their study.  Data/Methods   - Case studies🡪 survey Qs: The process through which the authors create variables off the case studies (these are not surveys, right?) should also be more clearly stated. - Selection bias in the sample: It is unclear how the authors deal with selection bias in their survey/case studies. Line 248 states that sample is drawn to balance gender and ethnicity. But was the sample chosen at random (stratified based on gender and ethnicity)? - The paper needs a description of how the variables are formulated, from what questions. - RE estimation (regression, shown in the formula 1)   The outcome variables need to be better explained. What is the difference between FNPF-1 and FNPF-2? What are the ‘benefits’ of which respondents are aware in FNPF-2? Are these the expected benefits farmers are going to collect (therefore testing the respondents’ ability to calculate their expected benefits)?  Lines 277-279 suggest that the authors did ask the respondents about all means of retirement savings –in addition to FNPF. This point should be clarified earlier.  **Sample size** **power issue**: The authors utilize a sample size of 71 respondents. This may be too small to generate statistically robust results for a regression with 8 regressors(independent variables). I suggest the authors conduct a power analysis to ascertain the minimum a-priori sample size for the regression (Faul et al. 2009). Using an online calculator, the minimum sample size the authors need for a regression with 8 regressors (with a moderate effect size of 0.15 and minimum statistical power level 0.8 (standard)) is 108 respondents.  <https://www.danielsoper.com/statcalc/calculator.aspx?id=1>  Faul, F., Erdfelder, E., Buchner, A., & Lang, A. G. (2009). Statistical power analyses using G* Power 3.1: Tests for correlation and regression analyses. Behavior research methods, 41(4), 1149-1160.  Findings   - It would be great if the authors could show a table of descriptive stats. - RE “94% of respondents prefer a tailor-made retirement plan”🡪 This statement does not really add to the paper. What are the plan specifics that they desire? To start discussing these, the authors should provide a more comprehensive and critical evaluation of the FNPF in light of its (non)coverage of informal sector workers. What are the FNPF’s shortcomings, and are these what the respondents want in the ‘tailored plan’? - RE line 332-333 “This contrasts the duration the person has been a market vendor.” This needs to be explained better. - RE lines 333-335 “Other savings avenues have a positive effect….financial literacy enables one to be aware of FNPF’s services.” This is a possibility but not the literacy is not the only facilitator that incentivizes a farmer to have other savings. The bank accounts may be a joint account for the family. It could just mean the respondents are wealthier and are in need of a secure means to safeguard their finances.      - **Interpretation of the findings in Table 3 and Table 4:**   The points of convergence and divergence in terms of the effects of the drivers in Table 3 and Table 4 should be explained. For instance, why is gender a significant predictor for awareness of the FNPF benefits (table 4) but not general awareness of FNPF (table 3)?  Line 341-342: While gender is a weak predictor of awareness of the pension schemes, the sample doesn't necessarily suffer from 'lack of awareness of FNPF' generally. The summary stats showed that 60% know FNPF and nearly 70% are aware of the benefits of FNPF right? The authors need to interpret findings more carefully.  From the earlier description in formula (1), AVNE was coded as a categorical variable (saving venues). But in Table 3 and Table 4, I only see one variable AVNE in the regression. How is this possible?   - What's the rationale behind doing multiple estimations--correlation, regression, principal component analyses? Rationales are not given in the paper.   Conclusion   - Lines 400-401: The fact that 94% of the respondents prefer ‘tailored pension’ deserves more elaboration. What kinds of design improvements are necessary? To make such recommendations, the authors need to critically evaluate the shortcomings of FNPF (for informal workers/farmers) in the background section. - The implications of the findings often do not align with their findings. For instance, in lines 404-405, the authors state that “FNPF needs to create more awareness for membership.” However, the awareness of FNPF in the sample was high—approximately 70%. This is quite high, compared to the level of understanding of other pension programs in other countries. I cannot agree that the lack of awareness is one of the main findings of this paper. - While financial literacy is indeed an important component of retirement planning (and a hot topic these days), I am not sure whether there is value in emphasizing the role of financial literacy this much in the paper. The authors are not really tackling financial literacy in this paper, based on the ways financial literacy is defined and measured and studied in the recent literature. ‘Awareness' and ‘financial literacy’ are two different concepts.   **REFERENCES**   - Faul, F., Erdfelder, E., Buchner, A., & Lang, A. G. (2009). Statistical power analyses using G* Power 3.1: Tests for correlation and regression analyses. Behavior research methods, 41(4), 1149-1160. - Ginneken W. Social Security for the informal sector: a new challenge for the developing countries. Int Soc Secur Rev. 2002;52:49-69. https://doi. org/10.1111/1468-246X.00033 - Hu YW, Stewart F. Pension coverage and informal sector workers: international experiences. OECD Publishing, 2009. Available from: <https://ideas.repec.org//p/oec/dafaab/31-en.html> - Lee, Z., Aguila, E., & Wong, R. (2023). Determinants of retirement of formal and informal sector workers in Mexico: the role of health and economic security. salud pública de méxico, 65(5), 434-445. <https://doi.org/10.21149/15178> - Onyango C, Olunga C, Oleche MO. Attitude of informal sector workers towards saving for retirement. IJNRIS. 2016;3(4):12-9 https://www.noveltyjournals.com/upload/paper/ ATTITUDES%20OF%20INFORMAL%20SECTOR-752.pdf - Tran C, Jung J. Transfers and labor market behavior of the elderly in developing countries: theory and evidence from Vietnam. New York: 2009 https://doi.org/10.2139/ssrn.1147649 | We thank the reviewer for this comment.  We thank the reviewer for this comment.  We thank the reviewer for this comment.  This has been addressed. We have removed boosting food production and increasing productivity as the justifications.  We thank the reviewer for this comment.  We mention the need for financial inclusion as a requirement for farmers to see farming as a long-term option.  We thank the reviewer for this comment.  Based on all the recommended changes, the LR is now connected.  We thank the reviewer for this comment.  We have added clear motivations based on reviewer comments.  We thank the reviewer for this comment.  We agree with the reviewer and have decided to exclude the PCA from the paper. We have included the process to create variables. Paragraphs 4, 5 and 6 of Methods section.  We thank the reviewer for this comment.  We have done our best to bring clarity to the introduction.  We thank the reviewer for this comment.  We have included a background on the FNPF in the introduction section.  All details are included as suggested by the reviewers.  We thank the reviewer for this comment  We have included statistics from 2022 Annual Report of FNPF in the introduction section, part of the superannuation of Fiji.  We thank the reviewer for this comment.  We have included a detailed explanation in the introduction with all details suggested by the reviewer comments.  We thank the reviewer for this comment.  We have included the definition of informal workers as well as their eligibility to retirement benefits.  We thank the reviewer for this comment.  We have included the definition for all nest eggs savings and superannuation.  We thank the reviewer for this comment.  Motivations that were based on incentives for food production or increasing productivity have been replaced with the decline of the agriculture industry and the need to financial inclusion of informal sector workers such as farmers.  We thank the reviewer for this comment.  As per the earlier comment, we are not following this narrative. We have included the micro-level policies that benefit informal workers.  We thank the reviewer for this comment.  We have two research questions and 3 objectives. We have rephrased/aligned the 3 objectives for clarity.  We thank the reviewer for this comment.  We have re-written the parts to address the suggestion.  We thank the reviewer for this comment.  We have removed the two sentences that discussed the findings.  We thank the reviewer for this comment.  The Literature Review does not have a section on its own therefore we have removed the Heading and sub-headings and tried to narrate it better.  We thank the reviewer for this comment.  We have included a new paragraph that includes all the literature mentioned by the reviewers.  We thank the reviewer for this comment.  We have included the relationship (and why we have included financial inclusion) between the informal sector and financial inclusion here.  We thank the reviewer for this comment.  We have re-aligned the section and justified the reason for using financial planning.  We link financial planning to retirement planning.  We thank the reviewer for this comment.  We link the lack of financial planning to lack of financial literacy. As one of our questions in the study are on awareness of saving in a superannuation fund and the awareness of the benefits of savings in a superannuation fund.  We have removed the determinants of financial literacy.  We thank the reviewer for this comment.  We have re-done this section and removed information that was not relevant.  We thank the reviewer for this comment.  We have moved this part to the first section, informal sector and financial planning. We have included only relevant investment opportunities for Fijian farmers.  We thank the reviewer for this comment.  The way variables are created from the survey questions is stated under the methods sections. Lines 261 to 281.  We thank the reviewer for this comment.  Convinience Sampling was used to select market vendors.  We thank the reviewer for this comment.  The way variables are created from the survey questions is stated under the methods sections. Lines 261 to 281.  We thank the reviewer for this comment.  We thank the reviewer for this comment.  We have included the distinction between FNPF-1 and FNPF-2. We have also described what we mean by benefits.  We thank the reviewer for this comment.  We thank the reviewer for this comment.  We have addressed this in the paper as a limitation.  We thank the reviewer for this comment.  This has been referenced.  We thank the reviewer for this comment.  Descriptive Statistics table has been added in Findings.  We thank the reviewer for this comment.  Critical evaluation and suggestion mentioned.  We thank the reviewer for this comment.  This has been explained.  We thank the reviewer for this comment.  We have tried to rephrase and address this suggestion and offer financial literacy as a possible explanation for this relationship.  We thank the reviewer for this comment.  We thank the reviewer for this comment.  We agree with the reviewer and have corrected our statement.  We thank the reviewer for this comment.  We have addressed this in the paper.  We thank the reviewer for this comment.  Rationales are added in the methodology section.  We thank the reviewer for this comment.  This has been addressed as per the comment above.  We thank the reviewer for this comment.  We agree, we have phrased the lack of intention to adopt FNPF as a savings avenue. | Agricultural sector development is one of the most powerful tools to end extreme poverty, boost shared prosperity, and feed around 10 billion people by 2050. Compared to other sectors, its growth is two to four times more effective in increasing the incomes of the poorest. Agriculture accounts for 4% of the global gross domestic product and more than 25% in the least developing countries [1]. Therefore, it is vital to protect and improve the welfare of workers in the agriculture sector. This sector mainly contains informal workers who are excluded from social safety nets and government protection programmes. Based on the right to decent work and job security, informal workers should be provided protection and social safety. This will not only retain current farmers but also attract new and young farmers to look at agriculture as a career choice. A typical financial and social security instrument is superannuation. While workers from formal sectors have access to superannuation, informal workers do not. Similarly, in Fiji, formal sector workers and employers make mandatory contributions to superannuation funds. However, this is only an option for informal sector workers such as farmers.  Additionally, The Reserve Bank of Fiji states a need for financial inclusion policies by the government to incentivise financial products and models aimed at financially excluded populations [5], such as farmers who do not have access to superannuation, which is a deterrent for them to consider farming as a long-term career.  In 2022, there were a total of 398,593 members in FNPF, 214,536 of whom were compulsory members while 12,242 were voluntary members. Out of all voluntary members, 55% were females and 45% were males. There were 6,632 voluntary members in 2021 compared to 12,242 in 2022. Voluntary members contributed 4.8 million in 2021 and 20.1 million in 2022.  Informal workers include those who are unskilled and those who are self-employed. Informal workers in Fiji include farmers, market vendors, domestic workers, those hired on short-term contract work such as landscaping, carpenters, construction workers among those hired on daily or short-term basis for different tasks.  Nest Eggs savings are savings and investments for huge financial goals such as retirement.  Superannuation is a fund that workers regularly contribute towards a future pension.  This motivation is based on the fact that from the early 1960s till the late 1900s, the agriculture sector outweighed the manufacturing and service sectors.  Additionally, The Reserve Bank of Fiji states a need for financial inclusion policies by the government to incentivise financial products and models aimed at financially excluded populations [5], such as farmers who do not have access to superannuation, which is a deterrent for them to consider farming as a long-term career.  The Fiji government provides six main social assistance programs. These include the poverty benefit scheme, care and protection allowance, social pension scheme, disability allowance, rural pregnant mother’s food vouchers and the bus fare scheme  The survey design is intended to answer our primary research questions: Is there a savings plan for small market vendors? Do these small market vendors have a mapped retirement plan for old age? Consequently, the objectives of the current study are set as follows: (1) to establish whether vegetable market vendors in the western division are aware of savings with FNPF; (2) to establish whether vegetable market vendors in the western division are aware of the benefits of saving with FNPF; and (3) establish whether vegetable market vendors of western division prefer a tailor-made superannuation savings scheme.  Financial planning and, specifically, retirement planning in agriculture, is important because it is a crucial step that brings economies closer to achieving the United Nations' sustainable development goals reducing poverty, providing decent work, and reducing inequalities for informal workers such as farmers  The aforementioned studies indicate a lack of financial inclusion especially in developing countries. Therefore, it is imperative to understand the importance of financial inclusion.  Given the difficulties in gathering enough respondents for analysis, the study used a convenience sampling technique, where participants were recruited based on whoever was willing to participate (Sears, 1986). Convenient sampling was used for data collection as market vendors fall under the administration of local municipal councils whose approval needs to be sought before data collection in their respective markets. This was the case in our research, where approval was not always granted by municipal councils to interview market vendors. As such, we were only given permission to interview three markets in the western division of Fiji and were advised by the market administrators to approach market vendors for interviews only if they are willing to participate. Even for markets where we received permission to interview market vendors, market vendors themselves were reluctant to be part of the interviews. For this reason, convenient sampling was adopted.  The key distinction between the two constructs is that FNPF-1 measures whether market vendors are aware of FNPF as an organization that provides superannuation services. FNPF-2 on the other hand measures whether individuals are aware of the benefits FNPF provides. These benefits include pension oon retirement, annual accumulating interest, housing assistance, education assistance among other benefits provided by FNPF to its members. To understand whether market vendors are aware of the benefits of FNPF, it is important to first understand whether they are aware of FNPF as an organization. 62% of market vendors are aware of FNPF as a savings avenue while about 67% are aware of the benefits of savings with FNPF.  Specifically, the sample size is a bit small for regression analysis. A power analysis for a regression with 8 regressors with a moderate effect size of 0.15 and minimum statistical power of 0.8 returns 108 respondents [67].  The current FNPF structure is a pro-formal sector where employees and employers have a portion of their income and contribution by the employer directly deposited into an employee’s FNPF account. This does not suit informal sector workers such as market vendors because their income fluctuates, and there are no other formal avenues in which they can easily and flexibly contribute to their FNPF account like their formal sector counterparts. This tailor-made plan needs to be flexible and easy for informal sector workers to contribute to. One way this can be achieved is through the only formal payment that market vendors make, the market vendor fees that are paid to the municipal council. The respective stakeholders such as FNPF, respective ministries and local municipal councils, can liaise and add an optional or fixed contribution to the market vendor fees market vendors will easily be able to contribute. After collection of the fee, local municipal councils can credit the amount to the FMPF account of respective market vendors. This creates a simple, flexible and easy way for market vendors to make FNPF contributions.  This indicates that the older the market vendor, the less aware they are of savings with FNPF and the benefits of savings with FNPF.  Other savings avenues have a positive effect which means that the more the market vendors have knowledge of other savings avenues, the more they are likely to know about FNPF as a savings avenue. This may indicate that the more financially literate individuals are, the more they are likely to know about FNPF as a savings avenue.  Relating this to the measure of gender employed in this study, one explanation for this inconsistent finding is that women may not understand what FNPF exactly does as an organisation but are aware that it is beneficial. In other words, there appears to be a gap in their knowledge regarding the specific function FNPF plays in the Fijian economy, although they know it is beneficial.  Generally, about 62% of respondents are aware of savings with FNPF, while about 68% are aware of the benefits of saving with FNPF. This indicates a good awareness level of market vendors in terms of savings awareness and benefits of savings awareness with FNPF. This is contrary to the findings of Gough and Hick [44], Loretto et al. [45] and Heenkenda [54].  Correlation analysis was used to determine whether there are any positive or negative relationships between our variable that could provide us with meaningful relationships. The regression analysis was used to determine factors that determine awareness of savings with FNPF and factors that determine awareness of savings benefits with awareness. They were tested separately to understand whether there are any significant differences between awareness of savings with FNPF and awareness of the benefits of savings with FNPF. |
| Journal Requirements:  1. Please ensure that your manuscript meets PLOS ONE's style requirements, including those for file naming. The PLOS ONE style templates can be found at  <https://journals.plos.org/plosone/s/file?id=wjVg/PLOSOne_formatting_sample_main_body.pdf> and  <https://journals.plos.org/plosone/s/file?id=ba62/PLOSOne_formatting_sample_title_authors_affiliations.pdf>  2. Note from Emily Chenette, Editor in Chief of PLOS ONE, and Iain Hrynaszkiewicz, Director of Open Research Solutions at PLOS: Did you know that depositing data in a repository is associated with up to a 25% citation advantage (<https://doi.org/10.1371/journal.pone.0230416>)? If you’ve not already done so, consider depositing your raw data in a repository to ensure your work is read, appreciated and cited by the largest possible audience. You’ll also earn an Accessible Data icon on your published paper if you deposit your data in any participating repository (<https://plos.org/open-science/open-data/#accessible-data>).  3. In the ethics statement in the Methods, you have specified that verbal consent was obtained. Please provide additional details regarding how this consent was documented and witnessed, and state whether this was approved by the IRB  4. We note that your Data Availability Statement is currently as follows: All relevant data are within the manuscript and its Supporting Information files.  Please confirm at this time whether or not your submission contains all raw data required to replicate the results of your study. Authors must share the “minimal data set” for their submission. PLOS defines the minimal data set to consist of the data required to replicate all study findings reported in the article, as well as related metadata and methods (<https://journals.plos.org/plosone/s/data-availability#loc-minimal-data-set-definition>).  For example, authors should submit the following data:  - The values behind the means, standard deviations and other measures reported; - The values used to build graphs; - The points extracted from images for analysis.  Authors do not need to submit their entire data set if only a portion of the data was used in the reported study.  If your submission does not contain these data, please either upload them as Supporting Information files or deposit them to a stable, public repository and provide us with the relevant URLs, DOIs, or accession numbers. For a list of recommended repositories, please see <https://journals.plos.org/plosone/s/recommended-repositories>.  If there are ethical or legal restrictions on sharing a de-identified data set, please explain them in detail (e.g., data contain potentially sensitive information, data are owned by a third-party organization, etc.) and who has imposed them (e.g., an ethics committee). Please also provide contact information for a data access committee, ethics committee, or other institutional body to which data requests may be sent. If data are owned by a third party, please indicate how others may request data access.  5. Please include your full ethics statement in the ‘Methods’ section of your manuscript file. In your statement, please include the full name of the IRB or ethics committee who approved or waived your study, as well as whether or not you obtained informed written or verbal consent. If consent was waived for your study, please include this information in your statement as well.  6. Please ensure that you refer to Figure 2 in your text as, if accepted, production will need this reference to link the reader to the figure.  7. Please include your tables as part of your main manuscript and remove the individual files. Please note that supplementary tables (should remain/ be uploaded) as separate "supporting information" files | We thank the Editor for this suggestion.  We have done our best to comply with the journal style requirements.  We thank the Editor for this suggestion.  We have submitted the raw data.  We thank the Editor for this suggestion.  We have included the name of the committee that has approved the study.  We thank the Editor for this suggestion.  Yes.  We thank the Editor for this suggestion.  There are no ethical or legal restrictions.  We thank the Editor for this suggestion.  We have included this.  We thank the Editor for this suggestion.  We have referenced figure 2.  We thank the Editor for this suggestion.  We have included the tables in the main manuscript. | Ethics approval was sought from the University of Fiji Research Committee. Verbal consent was sought from market vendors before interviewing them. |
