# Supplementary material for: Informal workers’ perceptions of retirement planning in developing countries
Source: PLoS One. 2025 Apr 15;20(4):e0321214. doi: 10.1371/journal.pone.0321214 (PMC11999124; doi:10.1371/journal.pone.0321214)
Supplement: Attachment — Submitted filename: Response to Reviewers Revision 4.docx [file pone.0321214.s008.docx]

**Reviewer Response Document Revision 4**

| **Reviewer Comments** | **Author(s) Comments** | **Changes** |
| --- | --- | --- |
| Journal Requirements:  Please review your reference list to ensure that it is complete and correct. If you have cited papers that have been retracted, please include the rationale for doing so in the manuscript text, or remove these references and replace them with relevant current references. Any changes to the reference list should be mentioned in the rebuttal letter that accompanies your revised manuscript. If you need to cite a retracted article, indicate the article’s retracted status in the References list and also include a citation and full reference for the retraction notice. | We thank the Editor for this comment.  We have rechecked our references and ensured that it is complete. | Documented as Track Changes |
| Additional Editor Comments:  I would like to thank the authors for their hard work in revising the manuscript. As an action editor (guest editor) and a former reviewer in the first round, I have taken a thorough look at the past reviewers’ comments as well as the authors’ responses to the comments. Upon review, I have decided to accept the paper with a minor revision. Please see my comments below:  **1. Better highlight contributions:** In the introduction section, it would be great if the authors could explicitly state what the contributions of this paper is. What is new in this paper that the prior literature has not done? For instance, in line 123-124, the authors stated that they gained first-hand info on the market vendors’ view toward superannuation. Is this a first paper to do so in Fiji? | We thank the Editor for his comments. | The recommended changes have been completed, and the main contribution has been succinctly highlighted in the paper. Please see lines 124 to 128 in the revised manuscript. We reaffirm that this is the first study examining the determinants of FNPF and its benefits amongst informal sector workers such as market vendors in Fiji. |
| **2. Literature Review section:** Lines 134-142 are awkward. The authors say this: “It begins by reviewing literature on the association between the informal sector and financial literacy. It then reviews the literature on retirement planning, and superannuation schemes in developing countries. The link between these groupings is that informal sector workers may lack financial literacy.” 2-1. If this is the case, why not combine the three sections to center the discussion on the role of financial literacy? For instance, why not make a section named “informal sector, superannuation, and financial literacy”? The last two sections (“retirement planning” AND “superannuation in dev. countries”) could definitely be combined. 2-2. Try to weave in lines 137-142 inside the actual sections (i.e., informal sector and financial literacy, superannuation, or a combined section). 2-3. How does the current paper fit in the line of literature reviewed in the Lit Review section? How should the readers situate this paper, vis-à-vis all the papers mentioned in the Lit Review section? I would like to see some comments on this, before the authors move onto the Data section. | We thank the Editor for his comments.  We agree with the Editor’s constructive suggestion. | Lines 139-147 are deleted as it basically described what was being discussed in the various sections that follow.  2.1 We have combined the first two sections into one, “Informal sector, superannuation and financial literacy”.  We have also combined the last two sections into one, “Retirement planning and superannuation in developing countries”.  2-2. We have moved lines 142-147 towards the end of the section (before a background on Fiji) as it provides a sound summary and justification of the section (Lines 212-218).  2-3. We highlight the contribution of the current paper towards exiting Literature:  This study complements the literature on retirement planning in the informal sector. While awareness of superannuation funds has not been studied previously, our study shows good awareness of superannuation and superannuation benefits by Fijian market vendors. A distinction that has not been previously made but is vital in studying intentions to use superannuation was studied in this paper. Here, the distinction is made between being aware that there is a superannuation and knowing the benefits that the superannuation offers (we can call this financial literacy). This study suggests that awareness levels do not necessarily lead to the adoption of superannuation. This is mainly because existing superannuation plans are not simple and flexible enough for market vendors and informal workers in particular. Therefore, there is a need for a tailor-made scheme for market vendors and informal workers (Lines 318-327). |
| **3. Data and Methods Section – Objective 1:** Equation (1) and Equation (2)—lines 343-346—seem to be exactly the same except the outcomes. In such a case, please show only one equation with an outcome listed as ‘outcome’, and explain that the authors test two outcomes in separate regressions. 3-1. Please describe the variables used in complete sentences instead of a table (Table 1). It is more typical to describe them within the text—in a full paragraph. | We thank the Editor for his comments. | The recommended changes have been completed. Please see lines 368 to 381 in the revised manuscript. We have updated the formula to one equation. We have also added the descriptions of the variables in a paragraph and retained the table because it provides more detailed information on variable definitions. |
| **4. Data and Methods Section – Objective 2:** Please elaborate more on the ‘thematic analysis’ of the interview questions. What are the themes or the method of the analysis? | We thank the Editor for this comment.  We have described the process we have followed for the thematic analysis. | The thematic analysis is discussed in the results section. Codes were assigned to the responses to the interview questions. Upon coding all responses, the codes were used to determine underlying themes. The themes were reviewed to check if all codes had been included. The frequency of each of the themes is noted and denotes their importance (Lines 408-412). |
| **5. Results – Regression results (Table 4 & Table 5):** The authors need not show too many decimal points, or the z-statistics. It would be clearer if the authors could present coefficients, standard errors, and statistical significance in the form of asterisks next to the coefficients (For instance * if p<0.1, ** if p<0.05, *** if p<0.001). 5-1. Please streamline the tables. The authors don’t need two separate tables just to show two outcomes. These could be combined into one table. This way, readers will be able to compare the results (of the two outcome variables) more easily. | We thank the Editor for this comment. | We incorporated the suggestion and merged both tables into Table 4 in the revised manuscript. We have limited all numbers to 4 decimal places, indicated with asterisks which variables return statistically significant results, and re-organized the presentation of p-values. |
| **Minor 6. Line 46 – improve citation:** It is great to see more information on the size of the informal sector in Fiji. Please list the year of the cited stats, and if it is from too far back, please acknowledge in a footnote that more recent statistic is not available. | We thank the Editor for this comment.  We have included the year of the stat.  We have also included a footnote that a more recent stat is not available. | A more recent statistic on informal sector contribution to GDP is not available |
| **7. Sample – acknowledge limitations:** Please acknowledge in the Discussion Section that convenience sampling is used. What are some implications of doing so? For instance, would there be any potential selection bias in the sample that the authors could not address in the current study? It would be good to clearly acknowledge it as one of the limitations. | We thank the Editor for this comment. | We have mentioned convenience sampling in the results section as well (Line 417).  We have also included that convenience sampling is a limitation in this study as it may lead to potential selection bias. We have also included that future research should consider a representative sample (Lines 635-641). |
