# Supplementary material for: Informal workers’ perceptions of retirement planning in developing countries
Source: PLoS One. 2025 Apr 15;20(4):e0321214. doi: 10.1371/journal.pone.0321214 (PMC11999124; doi:10.1371/journal.pone.0321214)
Supplement: Attachment — Submitted filename: PONE-D23-21438_Review.docx [file pone.0321214.s005.docx]

**REFEREE REPORT**

**PONE-D-23-21438: Informal Workers’ Perceptions of Retirement Planning in Development Countries**

**Comments to the Authors**

This paper examines whether and to what extent informal sector workers (i.e., vegetable market venders, or farmers) in Fiji engage in retirement savings—primarily by evaluating their awareness and access to Fiji National Provident Fund (FNPF). In doing so, the authors deploy several empirical estimation strategies including the OLS regression, correlation analysis, and principal component analysis on a first-hand survey (case studies?) of 71 respondents. The authors find that most respondents regarded FNPF in a positive light, with 60-70% of the respondents being aware of the FNPF scheme and its benefits. Yet, 94% of them still preferred an alternative pension scheme that is better tailored for them. The authors also find that age, gender, years of experience (duration) in the current job, and availability of other venues for savings strongly predicted the likelihood of being aware of FNPF schemes.

While I see the value in the authors’ introducing a primary dataset on an underexplored group of informal agricultural sector workers in Fiji, I find several limitations in the overall framing (i.e., motivation for examining FNPF) of the research questions, estimation strategies, and interpretation of the results. Moreover, the authors equate ‘awareness’ of a government-sponsored pension/superannuation scheme (i.e., FNPF) with ‘financial literacy’—when these two are discussed as two distinct concepts in the current literature. I discuss my main concerns below, in no particular order.

**MAIN REMARKS**

- **Weak framing of the research questions:**
  - The framing of the paper, detailed in the Introduction, is weak. The authors start the paper with the need for an increase of world’s food production to fight global poverty and population growth. It is strange for me to see the authors linking ‘poverty’ and ‘population growth’ to ‘food production’. Poverty arises not because of unavailability of food (the global stocks) but primarily due to the unequal distribution/access/affordability of food around the world.

RESPONSE: We thank the reviewer for this comment. We have amended the framing and development of the research question in the updated introduction section. We have removed the motivating points relating to food production and argued instead, with appropriate citations from peer-reviewed journals that focus exclusively on economic issues in Pacific island economies, that vegetable market vendors in Fiji are informal sector workers who have no formal contracts, and that such workers do not contribute to superannuation programs such as FNPF. Addressing whether such informal workers are aware of superannuation programs such as FNPF is important because informal sector workers contribute an estimated 23% of Fiji’s GDP. However, such informal sector workers are underrepresented in superannuation schemes.

- The authors also use ‘(the need to boost) food production’ as a reason to care about the ‘welfare of food suppliers’. This is not wrong, but is this necessary? Without making these jumps, I feel like the paper can just start with a need to improve welfare for informal sector workers—who are often excluded from social safety nets and government protection programs, just based on the right to decent work and job security.

RESPONSE: We thank the reviewer for this comment. Such statements have been removed from the updated introduction.

- The authors’ justifying the need for FNPF and other retirement savings schemes of ag farmers based on the incentives they give for food production (i.e., increasing productivity) is awkward because retirement savings essentially provide annuities *after* retiring. Do retirement savings really provide ag farmers incentives to be more productive *during* their working years? The rest of the paper talks about retirement financial planning, so I am not sure whether this ‘boosting production’ framing is effective.

RESPONSE: We thank the reviewer for this comment. Such statements have been removed from the updated introduction.

- **Lacking flow**: The paper often lacks flow of narrative. For instance, the authors note in lines 66-69 in the Introduction that the main elements that contributed to the demise of the ag sector (and its production levels) were “property rights issues, preferential agreements, natural disasters, rural-urban migration, and lack of diversification.” None of these point to the lack of welfare or retirement benefits. So, if the authors were to start with the ‘need to boost food production’, the authors should be addressing existing government policies that deal with any of the key elements above—and not a pension scheme/retirement financing. Following the list of key elements that deterred production in the ag sector, the authors say that they study FNPF in this study (lines 71-73). At the moment, it is not clear why the discussion of the declining production in the ag sector calls for a need to study FNPF. How does this superannuation fund address the key elements of the demise?
  - Another instance can be found in the Lit Review section. The subsections are fundamentally related, but the authors don’t make enough effort to point out any connections.

RESPONSE: We thank the reviewer for this comment. This statement has been removed from the paper. We now make no mention of the declining agricultural productivity to motivate the paper.

- **Overall, motivation and justification for the authors’ exploration of FNPF (detailed in the paper) need a lot more polishing.**
- **There is no justification for several methods used in the paper.** For instance, what’s the value of using principal components analysis and regressions? **Data:** The process through which the authors create variables off the case studies (these are not surveys, right?) should also be more clearly stated.

RESPONSE: We thank the reviewer for this comment. We have attempted to polish and amend the motivation and justification of the paper.

**COMMENTS ON SPECIFIC SECTIONS**

Introduction

- **The scope of the paper can be made clearer**. For instance, the authors state that the paper address literature on ‘nest egg savings options. But isn’t the paper specifically centering on a type of contributory pension (Fiji National Provident Fund, or FNPF) and no other general saving schemes for non-retirement purposes? Why not just define the scope as looking at pension—and the extent to which ag farmers in Fiji rely on it?

RESPONSE: We thank the reviewer for this comment. We have attempted to better articulate the scope of the paper in lines 112-121 in the updated introduction. This paragraph also details the objectives of the study.

- **More background info on FNPF is needed in the introduction**: FNPF is not mandatory (line 31, 56), but what does that mean? FNPF seems to be a contributory pension scheme where employers match the workers’ contribution amounts, and this is mandatory for formal sector workers. For informal sector workers (i.e., ag farmers) for whom FNPF is not mandatory, what happens if an informal sector worker decides to contribute to FNPF? Does his/her employer (in the informal sector) need to match the amount? Or are they all considered self-employed workers?

RESPONSE: We thank the reviewer for this comment. More details on FNPF are provided in the updated introduction starting from line 50. Specific details on contribution rates and benefits are in lines 70-88.

- On a related note, what are the general retirement savings patterns in the Fiji informal sector? Any statistics or prior literature? What is the usual share of informal sector workers that rely on FNPF?

RESPONSE: The share of informal sector workers that rely on FNPF is unknown in Fiji. We have cited a relevant paper that explains the potential contribution of informal workers to the Fijian economy. This is cited in the first paragraph of the introduction and stands at 23%.

- In the introduction, there is no background on retirement savings patterns of individuals in Fiji, or FNPF. There is one paragraph of the background on FNPF (lines 223-234) in the Lit Review section—but it comes way too late. It may be better to put these earlier, as a part of the Introduction

RESPONSE: We thank the reviewer for this comment. The background information on FNPF has been moved to the introduction. This, we believe, succinctly explains savings patterns in Fiji.

- Definition of informal workers seems incomplete (line 51-52). What about government protections or tax or retirement benefits eligibility?

RESPONSE: The definition of informal sector workers is updated in lines 49-53 of the updated introduction.

- It may be helpful for general readers to define concepts such as ‘nest egg savings’ or ‘superannuation’ once in the introduction (lines 28, 30, 54).

RESPONSE: The definition of “nest egg savings” is done in lines 28-29. The definition of superannuation is in lines 62-63.

- Going back to the awkwardness of justifying the need for FNPF and other retirement savings schemes of ag farmers based on the incentives for food production (i.e., increasing productivity), this comes back to bite in lines 57-64. In discussing policy alternatives to FNPF, the authors discuss policy alternatives to boost food production—including export subsidies. Isn’t the main purpose of FNPF or any other pension schemes to promote welfare and retirement preparedness?
  - Even if the authors decide to keep the narrative linking FNPF to boosting food production/productivity, the discussion of policy alternatives is very limited in the paper (lines 57-64) and requires more nuanced descriptions. There exist several micro-level policies that directly benefit workers—such as living subsidies, childcare subsidies, or even food vouchers.

RESPONSE: We thank the reviewer for this comment. We have justified the need for FNPF based on the case that informal sector workers such as market vendors contribute about a quarter of Fiji’s GDP yet are underrepresented in superannuation programs. We have removed the need discussion on food production.

- Lines 74-81: The specific survey Qs seem to ask whether workers utilize FNPF account, and to what extent,, and characteristics that predict FNPF usage/awareness. The 3^rd^ question (line 78-79) should be more carefully worded. The authors seem to estimate determinants that ascertain ag farmers’ probability/likelihood of being aware of FNPF. This is the outcome variable the authors use. The extent of awareness and the extent of accessibility are two different concepts.
- I see another stretch of an argument when the authors link the value of ‘financial planning’ (at an individual level) to ‘UN’s sustainable development goals or reducing poverty or reducing inequalities (for whom?)’.

RESPONSE: We thank the reviewer for this comment. We have refrained from linking the motivation of the paper to the UN SDGs.

- Lines 86-93 should probably go to the discussion/conclusion section. The authors talk about implications of findings here, but they haven’t described their findings yet.

RESPONSE: These parts have been removed from the updated manuscript.

Literature Review/Background

- **Overall:** The background section has too many subsections without any narrative cutting through them. They are fundamentally related, but the authors don’t make enough efforts to point out any connections.

RESPONSE: Appropriate links between the sectors are in lines 134-140.

- **RE Informal Sector and Financial Inclusion**: Relevant studies on informality and retirement preparedness that are the authors missed include the following:
  - Ginneken W. Social Security for the informal sector: a new challenge for the developing countries. Int Soc Secur Rev. 2002;52:49-69. https://doi. org/10.1111/1468-246X.00033
  - Hu YW, Stewart F. Pension coverage and informal sector workers: international experiences. OECD Publishing, 2009. Available from: <https://ideas.repec.org//p/oec/dafaab/31-en.html>
  - Lee, Z., Aguila, E., & Wong, R. (2023). Determinants of retirement of formal and informal sector workers in Mexico: the role of health and economic security. salud pública de méxico, 65(5), 434-445. <https://doi.org/10.21149/15178>
  - Onyango C, Olunga C, Oleche MO. Attitude of informal sector workers towards saving for retirement. IJNRIS. 2016;3(4):12-9 Available from: https://www.noveltyjournals.com/upload/paper/ ATTITUDES%20OF%20INFORMAL%20SECTOR-752.pdf
  - Tran C, Jung J. Transfers and labor market behavior of the elderly in developing countries: theory and evidence from Vietnam. New York: 2009 <https://doi.org/10.2139/ssrn.1147649>

RESPONSE: These papers have been cited in the updated manuscript.

- **RE Informal Sector and Financial Inclusion** **cont.:** There is a jump in logic in line 102. Up to this line, the authors discuss that informal sector research is limited. But why is financial inclusion suddenly brought up here? For instance, are informal labor characterized by financial exclusion (from govt programs? retirement financial under-preparedness?)? Also, the citations included in line 102 and onward are no longer about the informal sector. It is more about the value of financial inclusion generally. This section needs to be woven better. Why discuss informality and financial inclusion together? How do the two relate?

RESPONSE: These parts have been amended to discuss financial literacy within the context of informal workers such as market vendors.

- **RE Financial Planning:** This section talks about financial planning generally and provides some details on superannuation (lines 121-). But given the overall topic of this paper (on informal sector workers), the authors should still tie this section to informal sectors. For instance, in what ways are superannuation schemes applied to informal workers? There is another sudden jump in line 140. Why the sudden discussion of retirement planning—when the prior discussions of financial planning in this section did not mention retirement planning at all?

RESPONSE: Appropriate links have been made to link these concepts in lines 134-140. We maintain the importance of retaining this section because retirement planning, which market vendors seem to lack in Fiji, is a topic nested within financial planning. As such, the financial planning literature, in our opinion, offers useful insights into this phenomenon. WE have amended the heading of this subsection to “retirement planning” to better reflect its contents.

- **RE Financial Planning cont.:** It is not clear why the authors discuss the link between retirement planning and financial literacy at length in this section (lines 140-). The authors need to provide clearer description as to why they are emphasizing financial literacy. For instance, are the authors empirically measuring financial literacy in their estimation? The authors go on to list determinants of financial literacy. These distract the readers. Are these drivers measured in the analysis? If yes, the authors should state this.

RESPONSE: Following the earlier comment of the reviewer, we have revised the entire literature review section to cohesion. For this purpose, we have decided to retain the discussion on financial literacy but have attempted to relate this to retirement planning, noting that the latter is the subject of the previous subsection. For the purpose of “weaving” the sections together, we feel that this is important.

- **RE Superannuation in Developing Countries:** This section summarizes findings on several different aspects of pension, from (1) lack of participation or access to pension in Indonesia, to (2) effect of pension on well-being. I am afraid these studies are summarized one after another without a clear rationale. I would say, for this paper, the main thing I need to know from the existing literature is how superannuation contributes to retirement planning or retirement financial security. OR how superannuation boosts productivity of participating workers. More importantly, the authors seem to discuss the pensions without delineating between different types of pension (annuity vs lump sum provision, contributory, non-contributory). The effects of pension on retirement planning, for example, depends on the pension type. So the authors should make efforts to acknowledge the distinctions.

RESPONSE: Discussing superannuation schemes in other developing countries helps situate our paper within the broader literature focusing on superannuation schemes in developing countries.

- **RE other investment opportunities for farmers:** Another floating section. This section can be woven together with the first background section (informal sector and financial planning), to show that farmers have alternatives for retirement savings: FNPF, or these other investment opportunities. Also, the authors need to better describe how this information is going to be used in their study.

RESPONSE: This has been removed and integrated into the introduction.

Data/Methods

- Case studies🡪 survey Qs: The process through which the authors create variables off the case studies (these are not surveys, right?) should also be more clearly stated.
- Selection bias in the sample: It is unclear how the authors deal with selection bias in their survey/case studies. Line 248 states that sample is drawn to balance gender and ethnicity. But was the sample chosen at random (stratified based on gender and ethnicity)?

RESPONSE: Please see lines 312-318. Convenience sampling was used.

- The paper needs a description of how the variables are formulated, from what questions.
- RE estimation (regression, shown in the formula 1)
  - The outcome variables need to be better explained. What is the difference between FNPF-1 and FNPF-2? What are the ‘benefits’ of which respondents are aware in FNPF-2? Are these the expected benefits farmers are going to collect (therefore testing the respondents’ ability to calculate their expected benefits)?
  - Lines 277-279 suggest that the authors did ask the respondents about all means of retirement savings –in addition to FNPF. This point should be clarified earlier.

RESPONSE: These issues are resolved in the updated Table 1 in the updated manuscript.

- **Sample size** **power issue**: The authors utilize a sample size of 71 respondents. This may be too small to generate statistically robust results for a regression with 8 regressors(independent variables). I suggest the authors conduct a power analysis to ascertain the minimum a-priori sample size for the regression (Faul et al. 2009). Using an online calculator, the minimum sample size the authors need for a regression with 8 regressors (with a moderate effect size of 0.15 and minimum statistical power level 0.8 (standard)) is 108 respondents.
  - <https://www.danielsoper.com/statcalc/calculator.aspx?id=1>
  - Faul, F., Erdfelder, E., Buchner, A., & Lang, A. G. (2009). Statistical power analyses using G* Power 3.1: Tests for correlation and regression analyses. Behavior research methods, 41(4), 1149-1160.

RESPONSE: While we thank the reviewer for bringing this to our attention, at this stage, it is infeasible to pursue this given funding and other administrative issues. We have highlighted this as a limitation to our current study in lines 603-606 of the updated conclusion.

Findings

- It would be great if the authors could show a table of descriptive stats.

RESPONSE: Please see Tables 2 and 3 in the revised manuscript.

- RE “94% of respondents prefer a tailor-made retirement plan”🡪 This statement does not really add to the paper. What are the plan specifics that they desire? To start discussing these, the authors should provide a more comprehensive and critical evaluation of the FNPF in light of its (non)coverage of informal sector workers. What are the FNPF’s shortcomings, and are these what the respondents want in the ‘tailored plan’?

RESPONSE: This is a policy outcome and an such, is now discussed in the conclusion section. Further details are elaborated there as well as in the thematic analysis.

- RE line 332-333 “This contrasts the duration the person has been a market vendor.” This needs to be explained better.
- RE lines 333-335 “Other savings avenues have a positive effect….financial literacy enables one to be aware of FNPF’s services.” This is a possibility but not the literacy is not the only facilitator that incentivizes a farmer to have other savings. The bank accounts may be a joint account for the family. It could just mean the respondents are wealthier and are in need of a secure means to safeguard their finances.
- **Interpretation of the findings in Table 3 and Table 4:**
  - The points of convergence and divergence in terms of the effects of the drivers in Table 3 and Table 4 should be explained. For instance, why is gender a significant predictor for awareness of the FNPF benefits (table 4) but not general awareness of FNPF (table 3)?
  - Line 341-342: While gender is a weak predictor of awareness of the pension schemes, the sample doesn't necessarily suffer from 'lack of awareness of FNPF' generally. The summary stats showed that 60% know FNPF and nearly 70% are aware of the benefits of FNPF right? The authors need to interpret findings more carefully.
  - From the earlier description in formula (1), AVNE was coded as a categorical variable (saving venues). But in Table 3 and Table 4, I only see one variable AVNE in the regression. How is this possible?
- What's the rationale behind doing multiple estimations--correlation, regression, principal component analyses? Rationales are not given in the paper.

RESPONSE: Regarding the 4 points above (including sub-points), the updated regression results are discussed, we have ensured that the sample is balanced throughout, and then re-run the regression. Noting the potential issues with ordinary least squares, we have utilized the robust least squares option instead. The discussion of the robust least squares approach is given in lines 357-366. We have retained the correlation analysis because it serves as a form of preliminary descriptive statistics. Regression analysis is used for the first objective of the paper. We agree with the reviewer and see no need for the principal component analysis. We have therefore removed this.

Conclusion

- Lines 400-401: The fact that 94% of the respondents prefer ‘tailored pension’ deserves more elaboration. What kinds of design improvements are necessary? To make such recommendations, the authors need to critically evaluate the shortcomings of FNPF (for informal workers/farmers) in the background section.

RESPONSE: Plausible tailor-made programs are discussed in lines 536 to 553 in the conclusion section. The drawbacks of FNPF have also been highlighted in the introduction section, namely, that informal workers such as market vendors do not have FNPF accounts, and that more active effort is not made from FNPF with the exception of voluntary contributions noting that market vendors work Mondays through to Saturdays and are unable to leave their stalls and deposit into FNPF.

- The implications of the findings often do not align with their findings. For instance, in lines 404-405, the authors state that “FNPF needs to create more awareness for membership.” However, the awareness of FNPF in the sample was high—approximately 70%. This is quite high, compared to the level of understanding of other pension programs in other countries. I cannot agree that the lack of awareness is one of the main findings of this paper.

RESPONSE: We apologize for this oversight. This is rectified in the revised manuscript in lines 554 to 557.

- While financial literacy is indeed an important component of retirement planning (and a hot topic these days), I am not sure whether there is value in emphasizing the role of financial literacy this much in the paper. The authors are not really tackling financial literacy in this paper, based on the ways financial literacy is defined and measured and studied in the recent literature. ‘Awareness' and ‘financial literacy’ are two different concepts.

RESPONSE: We have attempted to rectify this shortcoming in lines 134 to 142 in the updated manuscript where we draw appropriate links within these concepts.

**REFERENCES**

- Faul, F., Erdfelder, E., Buchner, A., & Lang, A. G. (2009). Statistical power analyses using G* Power 3.1: Tests for correlation and regression analyses. Behavior research methods, 41(4), 1149-1160.
- Ginneken W. Social Security for the informal sector: a new challenge for the developing countries. Int Soc Secur Rev. 2002;52:49-69. https://doi. org/10.1111/1468-246X.00033
- Hu YW, Stewart F. Pension coverage and informal sector workers: international experiences. OECD Publishing, 2009. Available from: <https://ideas.repec.org//p/oec/dafaab/31-en.html>
- Lee, Z., Aguila, E., & Wong, R. (2023). Determinants of retirement of formal and informal sector workers in Mexico: the role of health and economic security. salud pública de méxico, 65(5), 434-445. <https://doi.org/10.21149/15178>
- Onyango C, Olunga C, Oleche MO. Attitude of informal sector workers towards saving for retirement. IJNRIS. 2016;3(4):12-9 https://www.noveltyjournals.com/upload/paper/ ATTITUDES%20OF%20INFORMAL%20SECTOR-752.pdf
- Tran C, Jung J. Transfers and labor market behavior of the elderly in developing countries: theory and evidence from Vietnam. New York: 2009 https://doi.org/10.2139/ssrn.1147649
